# One-Step Synthesis of N, P-Codoped Carbon Nanosheets Encapsulated CoP Particles for Highly Efficient Oxygen Evolution Reaction

**DOI:** 10.3389/fchem.2019.00805

**Published:** 2020-01-09

**Authors:** Yuchuan Liu, Xu Guan, Baobing Huang, Qiaohua Wei, Zailai Xie

**Affiliations:** Fujian Provincial Key Laboratory of Electrochemical Energy Storage Materials, College of Chemistry, Fuzhou University, Fuzhou, China

**Keywords:** one-step strategy, OER, CoP, carbon nanosheets, biomolecule

## Abstract

Oxygen electrocatalysis, especially oxygen evolution reaction (OER), is a central process during the actual application of rechargeable metal-air battery. It is still challenging to develop ideal electrocatalysts to substitute the commercial noble metal-based materials. In this work, we have constructed a new material, CoP nanoparticles, which are encapsulated by a biomolecule-derived N, P-codoped carbon nanosheets via a simple and facile one-step strategy. The as-prepared material releases a high electrocatalytic activity and stability for OER, with an overpotential of 310 mV to achieve 10 mA/cm^2^ in 1 M KOH. Importantly, we found that the phosphoric acid can not only introduce phosphorus dopant into 2D N-doped carbon nanosheets and play a role of pore-forming agent, but also participate in the formation of active center (cobalt phosphide). Moreover, the coverage of N, P-doped carbon can prevent the CoP nanoparticles from corrosion under the harsh reaction medium to achieve high and stable activity. We believe that our strategy can offer a novel pathway to synthesize new transition metal-based catalysts for electrocatalysis or other heterogeneous catalysis.

**Graphical Abstract:**
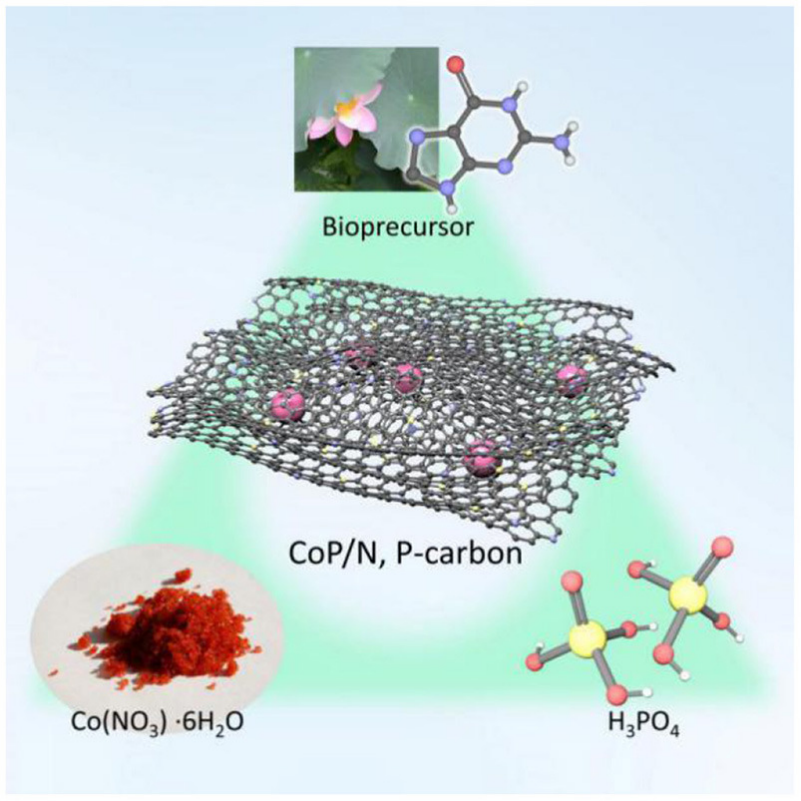
Bioprecursor-derived CoP/N, P-carbon material via one-step strategy.

## Introduction

The extensive application of renewable fuel cells and rechargeable transition metal-based air battery is an efficient and eco-friendly way to substitute traditional fossil fuels like petroleum and coal for energy demands (Guo et al., 2018; Huang et al., 2018; Miao et al., 2018). Among them, oxygen evolution reaction (OER) and oxygen reduction reaction (ORR) have drawn marvelous attention because of their high energy barrier of activating reactant and poor reaction kinetics, especially the OER process (Tan et al., 2017; Zhang et al., 2018; Hu et al., 2019b; Liu et al., 2019b). Generally, in order to facilitate the OER process, the noble metal-based catalysts, like RuO_2_ and IrO_2_, have been considered as high active electrocatalysts for OER (Li et al., 2018b; Teng et al., 2018). However, even though they exhibit high activities for electrocatalytic OER, the poor stability and high cost are two main reasons to impede their practical applications (Hu et al., 2018; Lei et al., 2018; Qiu et al., 2018). To deal with the poor stability of RuO_2_, recently, Shan et al. reported a nanocrystalline Ru@IrO_x_, where the high active site Ru is protected by IrO_x_, showing a high activity and stability for OER in acidic conditions (Shan et al., 2019). Unfortunately, the high cost of ruthenium and iridium is still an inevitable issue that is waiting for a better solution.

Recently, a great number of new materials that are effective for the OER process have been reported. Among them, transition metal phosphides, such as CoP_x_, have drawn a great deal of attention due to their high electrocatalytic activity and especially the low cost compared to the general RuO_2_ or IrO_2_ (Li et al., 2017; Fu et al., 2018; Liu et al., 2018). Thus, a great deal of effort has been made to design and synthesize CoP_x_-based materials with different nanostructures in order to further improve the actual performance (Xiao et al., 2017; Yan et al., 2017; Han et al., 2019; Li et al., 2019). Based on the previous reported works, the synthesis of CoP_x_ material generally consists of two processes: the formation of semi-finished product and the subsequent phosphorization at high temperature using Na_2_HPO_2_ as phosphor sources. For instance, Li et al. reported CoP/CoP_2_ nanoparticles encapsulated in N, P-doped CNTs via the decomposition of CoAl-LDH at high temperature and Na_2_HPO_2_ as phosphor source (Li et al., 2018a). The phosphorization via decomposition obviously complicates the preparing process and the release of PH_3_ gas is also poisonous. An ideal structure not only requires well-developed porous structure to promote mass transfer but also increases the electron conductivity (Chen et al., 2019; Jiang et al., 2019; Zhang et al., 2019). To access these, typical templates (hard or soft templates) are often required. Lin et al. used P123 as soft template to synthesize defective carbon–CoP hybrid material (Lin et al., 2018). Yuan et al. used CaCO_3_ as hard template to synthesize 3D carbon with *in situ* growing CoP nanoparticles (Yuan et al., 2016). From these previous works, it is obvious that although the template methods make the design of structure easier, the tedious processes impede the large-scale application.

In this work, we have successfully prepared a new 2D CoP-based carbon material via a simple and template-free one-step strategy. The CoP nanoparticles are encapsulated by 2D N, P-codoped carbon nanosheets that were derived from biomolecule guanine. The 2D nanosheet-like structure was clearly observed by an electron microscope. The N, P-doped carbon nanosheets not only provide a well-developed porous structure to promote mass transfer but also cover the CoP nanoparticles to preserve them from harsh conditions in alkaline electrolyte. The as-prepared material CoP-CGP2 exhibits an overpotential of 310 mV to achieve 10 mA cm^−2^ for OER in 1 M KOH. We believe that this work not only supplies a competitive electrocatalyst for OER but also opens a new pathway to design other kinds of catalysts.

## Experimental Section

### Synthesis of CoP-CGP

The N, P-doped carbon-covered CoP material was synthesized via a one-step strategy. Specifically, 2 g of guanine, 720 μL of H_3_PO_4_, and 30 mL of deionized water were added together into a 100-ml beaker and stirred for 30 min. Then, Co(NO_3_)_2_·6H_2_O was added. When forming homogeneous solution, the beaker was transferred into an oil bath and heated at 80°C to evaporate water. Finally, the precursor was transferred to a tube furnace and calcined at 1,000°C for 2 h under continuous N_2_ flow. The products were noted as “CoP-CGP1, CoP-CGP2, and CoP-CGP3” depending on the usage amount of Co(NO_3_)_2_·6H_2_O for 0.10, 0.20, and 0.30 g, respectively.

### Synthesis of Co-CG

The synthesis process of Co-CG is similar to that of CoP-CGP only without the addition of H_3_PO_4_.

### Synthesis of CGP

Specifically, 2 g of guanine was first mixed with 720 μl of H_3_PO_4_ and 30 ml of deionized water and then evaporated at 80°C. Finally, the guanine-based precursor was carbonized at 1000°C for 2 h under continuous N_2_ flow again.

### Synthesis of CG

The synthesis of CG is similar to that of CGP, just without the usage of H_3_PO_4_.

### Physical Characterization

Powder X-ray diffraction (XRD) was performed by RIGAKU Ultima IV, and diffraction patterns were attained by Cu Kα (λ = 1.5406 Å) radiation at a scanning of 10 min^−1^ from 5° to 80°. X-ray photoelectron spectroscopy (XPS) was conducted using ESCALAB 250. The Raman spectra were measured by Renishaw inVia with a 532-nm laser excitation. Surface area and porosity of materials were measured by Micromeritics ASAP 2020 plus and ASAP 2060. A field emission scan electron microscope (FESEM), Hitachi S-4800, was used, operating at 5.0 kV. A transmission electron microscope (TEM), FEI Talos F200s, was acquired, operating at 200 kV. The element analysis was done using an Elementar Vario EL.

### Electrochemical Tests

The electrochemical tests were conducted over a three-electrode cell where glassy carbon (GC, 4 mm), graphite rod, and Ag/AgCl electrode are the working electrode, counter electrode, and reference electrode, respectively. For sample preparation, 3 mg of sample, 280 μl of ethanol, 140 μl of deionized water, and 32 μl of Nafion (5%) were added together and subjected by ultrasonic treatment for 30 min to form homogeneous sample ink. Before the test, 12.8 μl of ink was dropped on the surface of GC, which had been polished before, and dried at 60°C. All electrochemical data were recorded by IviumStat multichannel electrochemical workstation (Ivium, Netherland). The OER performance was evaluated via linear sweep voltammetry (LSV) in 1 M KOH with iR compensated at a scan rate of 10 mV/s (the solution resistance was manually measured via EIS before LSV test). All of the potentials are converted to the reversible hydrogen electrode scale E (RHE) (V) = E (Ag/AgCl) + 0.1989 + 0.0591 pH. During the cycle voltammetry (CV) test, the potential range is 1.2–1.65 V (vs. RHE) and the scan rate is 50 mV/s. The electrochemical impedance spectroscopy (EIS) was recorded from 100,000 to 0.01 Hz at 10 mV amplitude potential.

## Result and Discussion

**Scheme 1:**
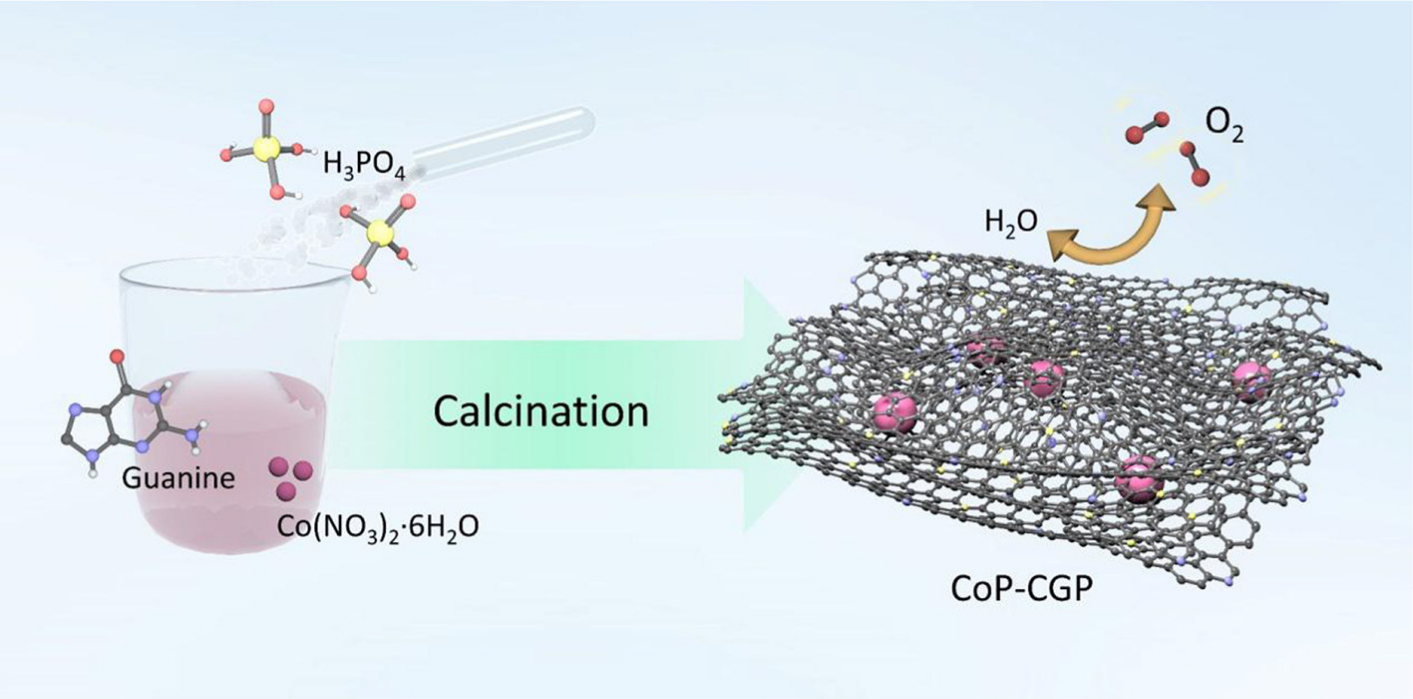
Illustration of the preparation process of the CoP-CGP material.

The CoP-CGP material was synthesized by means of a simple one-step strategy, as shown in Scheme 1. Typically, guanine, H_3_PO_4_, and Co(NO_3_)_2_·6H_2_O were dispersed homogeneously in 30 ml of deionized water. After evaporating the solvent, the mixture was directly carbonized at 1,000°C for 2 h under N_2_ flow. Guanine cannot merely *in situ* form the two-dimensional structure at high temperature but also introduce nitrogen atom into the carbon matrix (Huang et al., 2017, 2018). Moreover, the existence of phosphoric acid, which will decompose at high temperature, on one hand, participates in the formation of cobalt phosphide and, on the other hand, is *in situ* doped into the carbon matrix. In general, based on this simple and novel one-step preparation strategy, the CoP nanoparticles covered by 2D N, P-codoped carbon nanosheets can be successfully synthesized. The as-prepared materials were denoted as CoP-CGP1, CoP-CGP2, and Co-CGP3 according to the usage amount of Co(NO_3_)_2_·6H_2_O for 0.10, 0.20, and 0.30 g, respectively. The blank samples without H_3_PO_4_ and the pure guanine-derived N, P-doped carbon were denoted as Co-CG and CGP, respectively.

**Figure 1:**
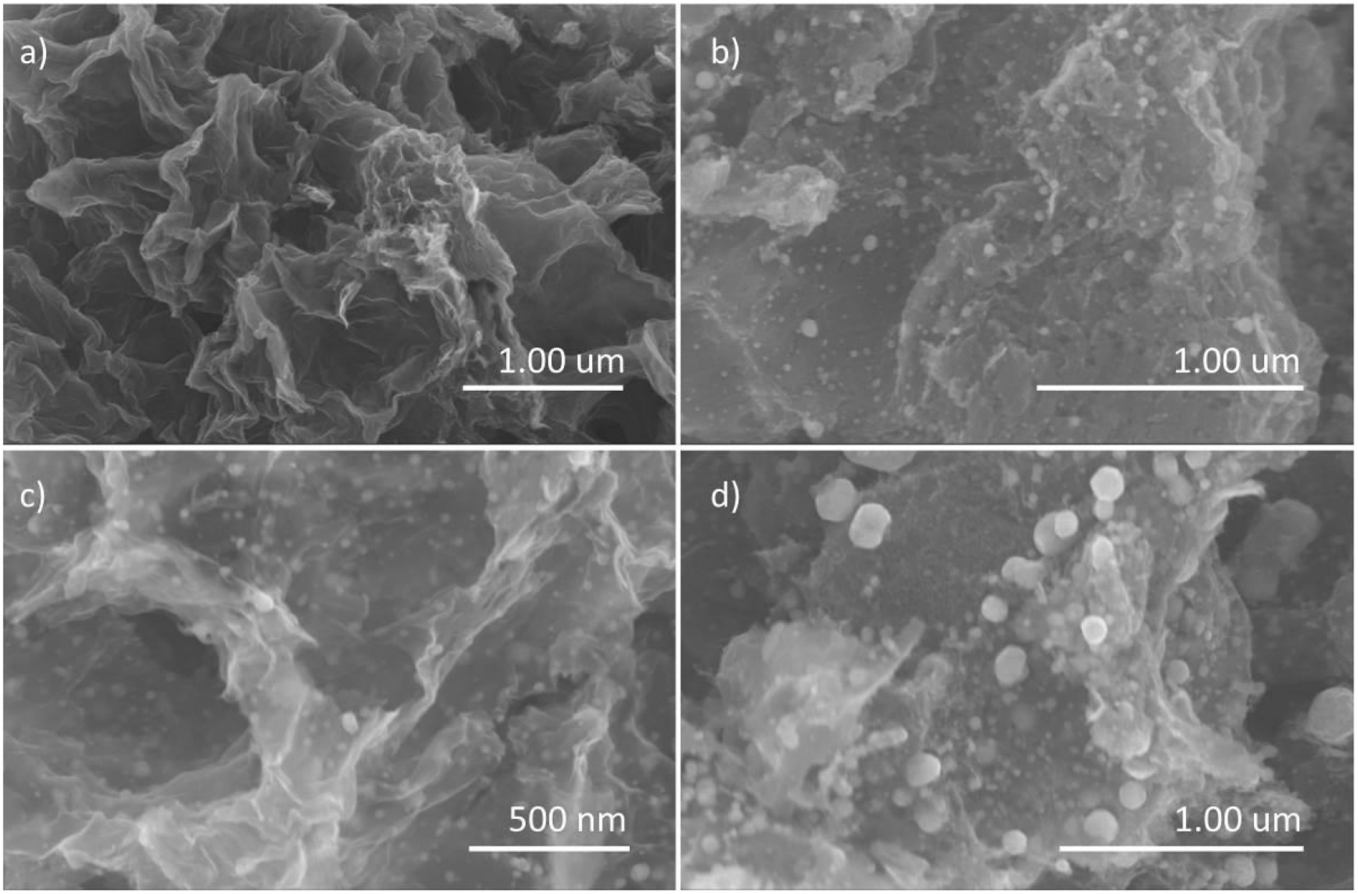
SEM images of **(a)** CGP, **(b)** CoP-CGP1, **(c)** CoP-CGP2, and **(d)** CoP-CGP3.

The morphology of the as-prepared materials was first observed via FESEM. As shown in Figure S1, the pure guanine-derived N-doped carbon, CG, displays a typical 2D nanosheet-like structure with wrinkles. When being carbonized in the existence of phosphoric acid, the *in situ* formed N, P-codoped carbon, CGP, still maintains the 2D structure as shown in Figure 1a, which suggests that the phosphoric acid will not impede the self-assembly of guanine to form 2D nanosheets. Moreover, the carbon nanosheets are transparent under the electron beam when observing the CGP by the TEM shown in Figure S2, which implies the thinness of those self-assemble 2D carbon nanosheets. The corresponding EDS elemental mappings (Figure S2) show the well dispersion of N and P on the surface of CGP.

**Figure 2:**
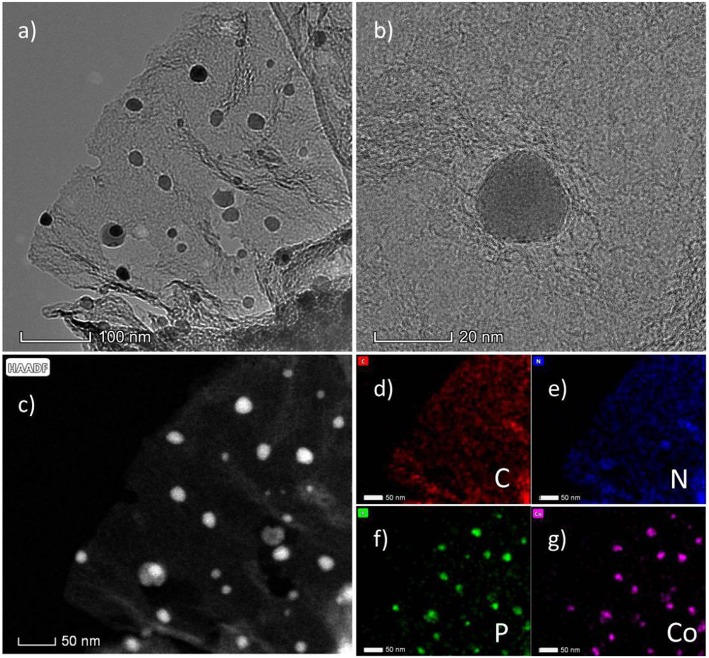
**(a,b)** TEM images of CoP-CGP2; **(c)** HAADF-STEM image and **(d–g)** EDS elemental mapping.

When introducing Co during the synthesis process, the CoP nanoparticles will be *in situ* formed during the carbonization process. As shown in Figures 1b–d, the SEM images exhibit the N, P-doped carbon nanosheets covering the CoP nanoparticles. Here, we synthesized three samples with different usage amount of Co(NO_3_)_2_·6H_2_O. As shown from the SEM images, the size of CoP nanoparticles increases with the increasing amount of Co(NO_3_)_2_·6H_2_O. CoP-CGP2 was chosen to be further observed under the TEM. As shown in Figure 2a, the thin nanosheet is transparent under the electron beam and decorated by CoP nanoparticles with a size of ~20 nm. In Figure 2b, it is noticeable that the CoP nanoparticle is covered by several carbon layers. The HAADF-STEM (Figure 2c) and EDS elemental mapping (Figures 2d–g) also show the dispersion of N and P. As the discussion aforementioned, the cobalt phosphide is generally considered as a competitive candidate because of its high activity to electrocatalysis. The coverage of heterogeneous doped carbon is expected to not only improve the electron conductivity but also play a role of protecting the CoP nanoparticle away from the harsh reaction condition.

**Figure 3:**
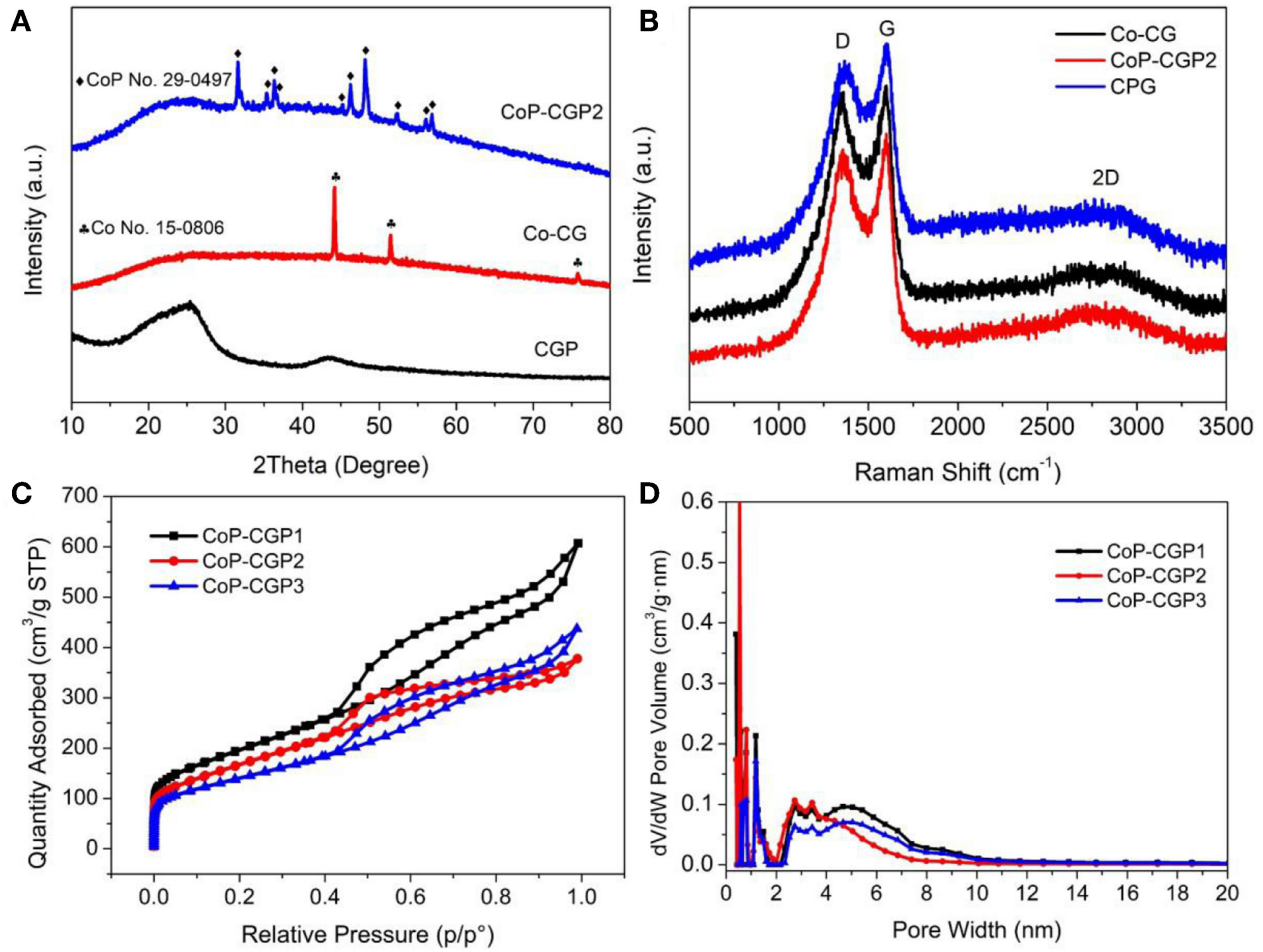
**(A)** X-ray diffraction (XRD) patterns; **(B)** Raman spectra; **(C)** N_2_ adsorption/desorption isotherms; and **(D)** pore size dispersion plots.

The one-step formation of CoP was further confirmed by X-ray diffraction pattern (XRD). As shown in Figure 3A, the CGP exhibits two broad diffraction peaks located at 26° and 42°, which correspond to the (002) and (100) planes of carbon material, respectively (Yuan et al., 2015; Hu et al., 2019a). When calcining the Co-based precursor, a broad peak is still shown at 26°. Besides, there exist several diffraction peaks that can be assigned to CoP (PDF No. 29-0497), suggesting the successful formation of CoP nanoparticles. For comparison, the blank sample synthesized without the usage of phosphoric acid was denoted as Co-CG. As shown in Figure 3A, the Co-CG displays diffraction peaks located at 44.2, 51.5, and 75.8°, which correspond to Co (PDF No. 15-0806). These results confirm again that the phosphoric acid not only plays the role in the formation of cobalt phosphide but also has little influence on the 2D nanosheet-like structure.

Raman spectra were then conducted and recorded. As shown in Figure 3B, three samples all show D band and G band located at 1,358 and 1,598 cm^−1^, respectively. The broad 2D band at around 2,600 cm^−1^ implies the sheet stack structure. Generally, the intensity ratio of D band to G band (*I*_*D*_/*I*_*G*_) is generally used to evaluate the graphitization and defect degree of carbon material (Qu et al., 2016; Liu et al., 2019a). We calculated and found that the Co-CG possesses the highest value of *I*_*D*_/*I*_*G*_of 0.97, suggesting a relatively higher graphitization degree. It may be attributed to the existence of Co, which plays a role of catalyst to facilitate the graphitization of guanine. Oppositely, the CGP shows the lowest *I*_*D*_/*I*_*G*_value of 0.92, suggesting a relatively higher defect degree. The value of CoP-CGP2 is 0.95, suggesting that the CoP-CGP2 keeps an ideal balance between graphitization and defect.

A N_2_ adsorption–desorption experiment at 77 K was conducted to explore the porous structure of samples. As shown in Figure 3C, three samples show similar isotherms. The presence of a sharp nitrogen uptake at low pressure is the monolayer filling of micropores. The following uptake between 0.10 and 0.40 is attributed to the initial few multi-layer adsorption on the external surface of mesopore or macropore. Finally, there is a hysteresis loop shown between 0.40 and 1.00. Hence, the three samples all show a micro-mesopore structure, which is also confirmed by the calculated pore size distribution (PSD) via nonlocal density function theory (NLDFT) method, shown in Figure 3D. From these results, the three samples all display micro-mesopore structures, which are beneficial to mass transfer. However, these samples possess different surface areas. Specifically, CGP shows the highest Brunauer–Emmett–Teller (BET) specific surface area of 871.9 m^2^/g, while Co-CG shows the lowest value of 234.5 m^2^/g (see Figure S3). It is noticeable that the phosphoric acid plays the role of pore-forming agent during the carbonization process. Benefiting from the H_3_PO_4_, the CoP-CGP2 shows a relatively high specific surface area of 609.9 m^2^/g. The addition of Co actually had an influence on the surface area. As shown in Table 1, the specific surface area decreases with increasing the usage amount of Co(NO_3_)_2_ and achieve 503.1 m^2^/g for CoP-CGP3. In Table 1, it clearly displays that the phosphoric acid can increase the microporous and mesoporous volumes, compared with those of Co-CG. These results confirm that the phosphoric acid not merely improves the specific surface area, but extends the micro-mesoporous volume, which will promote the mass transfer during catalysis.

**Table 1:** Textural properties and elemental compositions of various catalysts.

**Samples**	**Porosity**				**Elemental compositions, wt%**^a^		
	***S_***BET***_* [m^**2**^/g]**	***V_***total***_* [cm^**3**^/g]**	***V_***meso***_*[cm^**3**^/g]**	***V_***micro***_*[cm^**3**^/g]**	**C**	**H**	***N***
CoP-CGP1	705.6	0.703	0.103	0.580	77.74	0.94	4.26
CoP-CGP2	609.9	0.451	0.099	0.346	79.96	0.72	2.85
CoP-CGP3	503.1	0.482	0.070	0.404	84.92	0.85	3.29
Co-CG	234.5	0.399	0.026	0.309	74.60	0.56	2.18
CGP	871.9	0.830	0.658	0.134	77.03	1.39	3.22

a *Determined by element analysis (EA)*.

**Figure 4:**
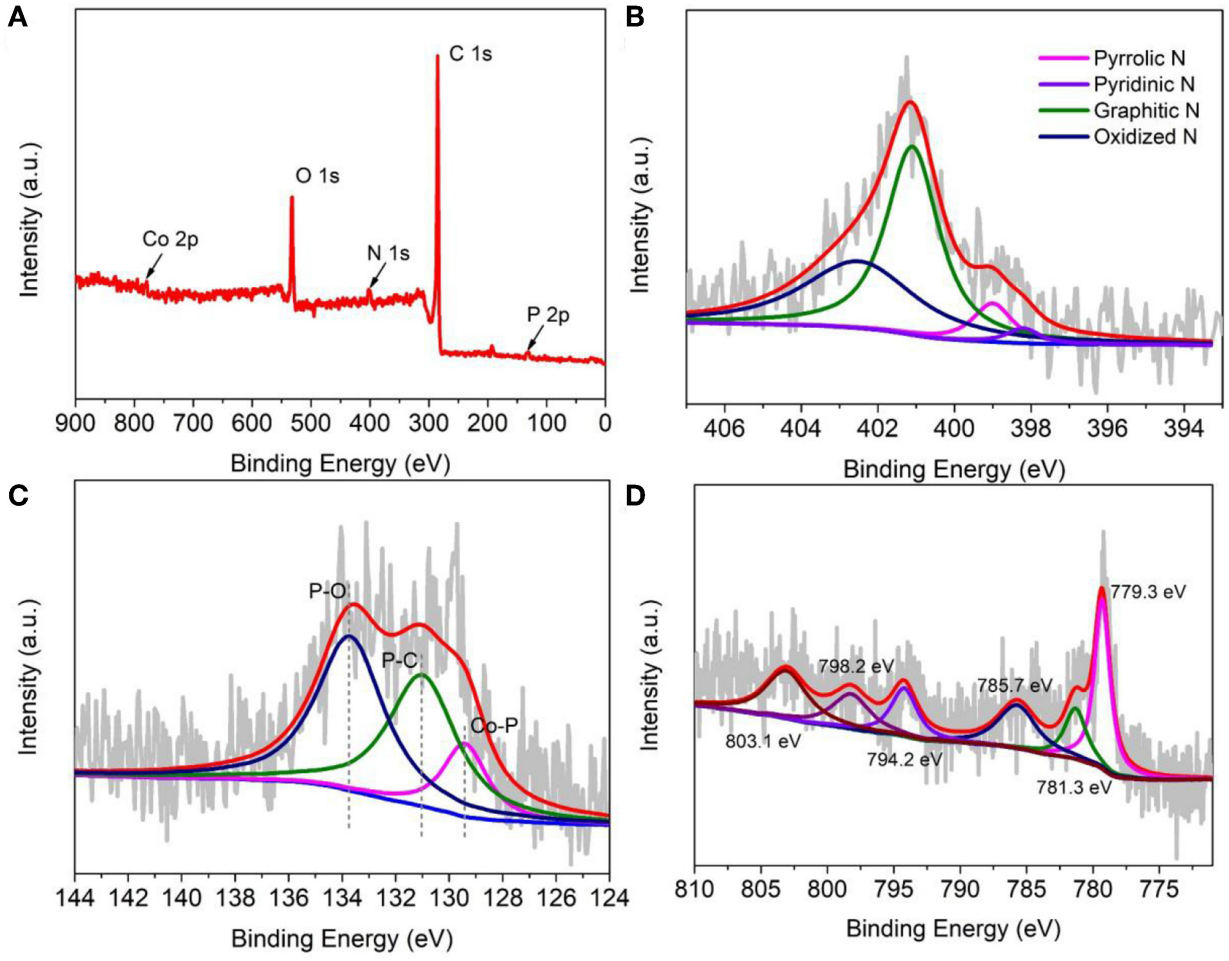
X-ray photoelectron spectroscopy (XPS) of CoP-CGP2: **(A)** survey; **(B)** N 1s; **(C)** P 2p; and **(D)** Co 2p spectra.

To further analyze the surface electron state of the as-prepared cobalt phosphide material, X-ray photoelectron spectroscopy (XPS) was conducted and recorded. Here, we representatively discuss CoP-CGP2, which shows much better electrocatalytic performance. From the survey spectrum as shown in Figure 4A, the as-prepared CoP-CGP2 material consists of five elements, i.e., C, N, O, P, and Co, which confirms again the successful doping of N and P. The N and P contents are 3.69 and 2.39 at%, respectively, based on the XPS data. For the high-resolution N 1s spectrum shown in Figure 4B, the fitted peaks at 398.2, 399.0, 401.1, and 402.5 eV assigned to pyridinic N, pyrrolic N, graphitic N, and oxidized N prove the doping of N atoms (Huang et al., 2019). The high ratio of graphitic N may be attributed to the introduction of Co, which catalyzes the graphitization of guanine. The high graphitic N will promote the electron conductivity of as-prepared material during the electrocatalysis process. For P 2p spectrum, the peaks at 131.0 and 133.7 eV of the P 2p spectrum (Figure 4C) can be assigned to P-C and P-O, respectively, suggesting the doping of P into the carbon matrix, while the peak at 129.4 eV corresponds to Co-P (Huang et al., 2018; Hou et al., 2019). Furthermore, as shown in the high-resolution Co 2p spectra (Figure 4D), the peaks at 781.3 eV (Co 2p 3/2) and 798.2 eV (Co 2p 1/2) can be assigned to Co^2+^. The peaks centered at 779.3 eV (Co 2p 3/2) and 794.2 eV (Co 2p 1/2) reflect Co^3+^. The other two peaks located at 785.7 and 803.1 eV are the satellite peaks, which correspond to the shake-up excitation of Co^3+^ (Li et al., 2018a). In short, the 779.3 eV of Co 2p and 129.4 eV of P 2p are ascribed to the binding energy of Co-P binding of CoP, which confirms again the formation of cobalt phosphide (Yang et al., 2019a).

## Electrochemical Tests

From the physical characterizations we discussed, we have successfully prepared CoP nanoparticles covered by 2D N, P-codoped carbon nanosheets, which is by means of a facile one-step strategy. Among the synthesis process, the addition of phosphoric acid not only participates in the formation of CoP and N, P-doped carbon, but also plays the role of pore-forming agent leading to a micro-mesoporous structure. Considering the active CoP sites and desirable structure, it can be expected that this as-prepared material will release a satisfying electrocatalytic performance.

**Figure 5:**
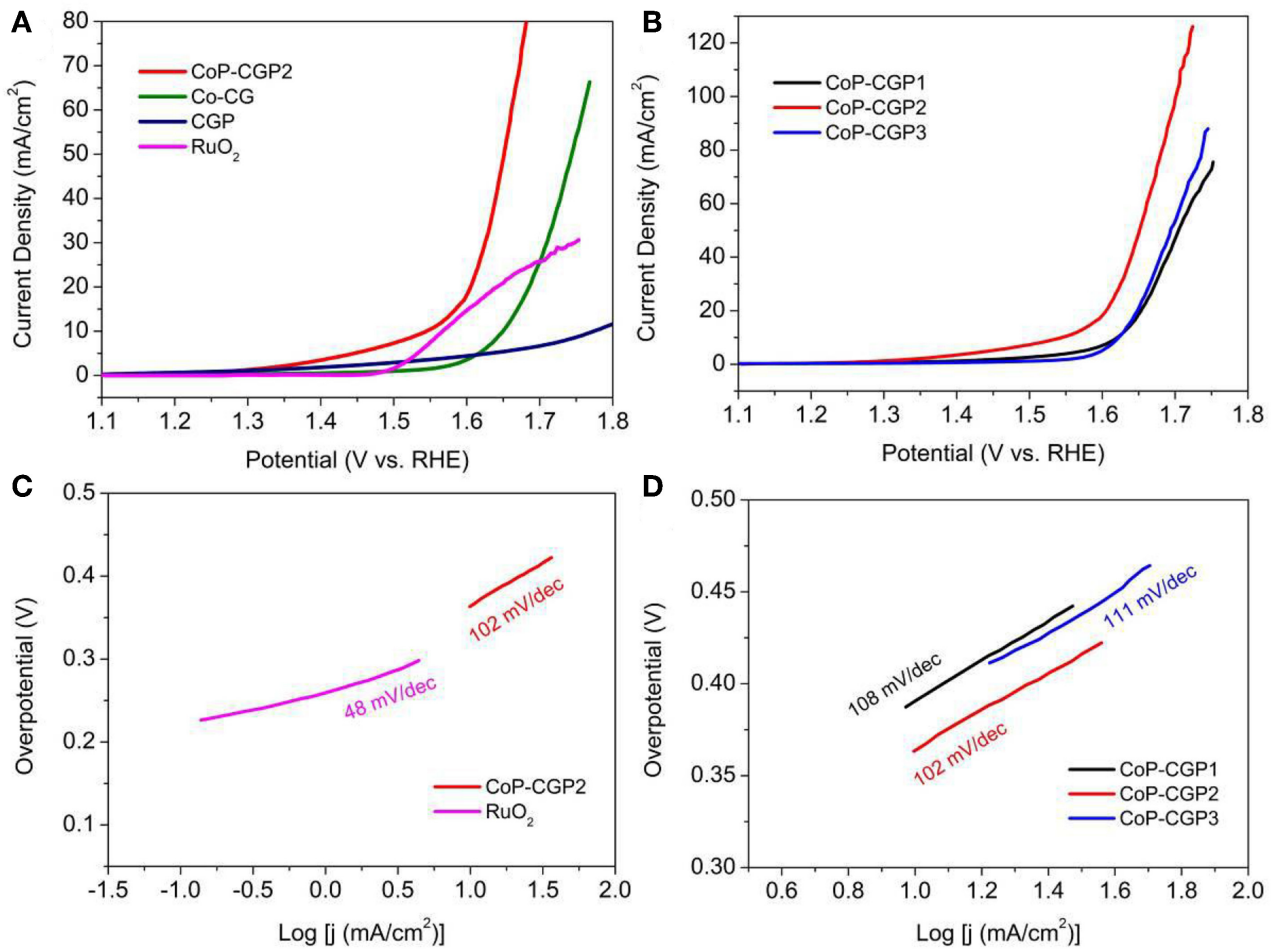
**(A,B)** Linear sweep voltammetry (LSV) curves in 1 M KOH; **(C,D)** Tafel plots.

Based on these considerations, we evaluated the OER activity of as-prepared materials. All the electrochemical tests were conducted in a typical three-electrode configuration cell in 1 M KOH solution. First, the activity was evaluated by means of LSV. As shown in Figure 5A, CGP displays the lowest OER activity with a high overpotential of 540 mV to release the current density of 10 mA/cm^2^. When adding Co during synthesis process, the obtained Co-CG exhibits an improved activity with a corresponding overpotential of 420 mV. For CoP-CGP2, this as-prepared CoP-based material shows the highest activity with a low overpotential of only 310 mV to drive the current density of 10 mA/cm^2^. It is obvious that the addition of phosphoric acid participates in the formation of CoP during the carbonization process, which is the OER active site; thus, the final obtained CoP-based material exhibits an ideal electrocatalytic activity. For comparison, the overpotential of commercial RuO_2_ to release 10 mA/cm^2^ is 330 mV, a bit larger than that of CoP-CGP2.

Furthermore, Tafel slope is another important parameter to analyze the reaction kinetics on the surface of catalyst. As shown in Figure 5C, the RuO_2_ shows the lowest value of only 48 mV/dec, suggesting the best OER reaction kinetics. The as-prepared CoP-CGP2 exhibits a relatively higher Tafel slope than that of RuO_2_, but lower than those of Co-CG and CGP, indicating a relatively ideal kinetics on the Co-CGP2. The effective amount of Co(NO_3_)_2_ was also explored. As the LSV curves shown in Figure 5B, when increasing the amount of Co(NO_3_)_2_, the activity of as-prepared CoP material was first increased and then decreased, wherein CoP-CGP2 achieves the best performance. Furthermore, CoP-CGP2 also exhibits the lowest value of Tafel slope (Figure 5D). Combined with the physical characterization, less Co(NO_3_)_2_ leads to higher specific surface area but less active CoP sites. Oppositely, more Co precursor will inevitably lead to agglomeration, which reduces the exposure of active sites. Thus, the moderate amount of the Co precursor is very important to achieve a good balance between specific surface area and CoP active site. In brief, the superior activity of CoP-CGP2 is likely attributed to the micro-mesoporosity property with relatively high specific surface area and ideal exposure of active CoP sites.

**Figure 6:**
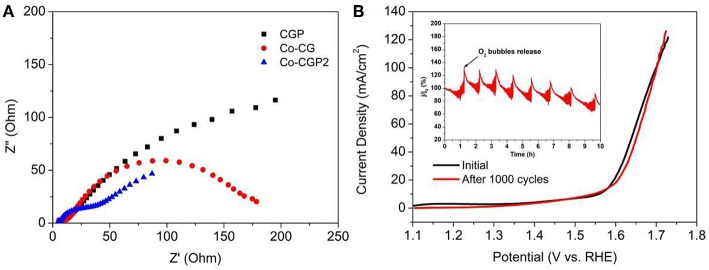
**(A)** Nyquist plots and **(B)** LSV curves before and after 1000 cycles (inset: current retention–time curve).

To further explore the high OER performance of this as-prepared CoP-based catalyst, the interfacial charge-transfer resistance (*R*_*ct*_) was measured by EIS. As shown in Figure 6A, the Nyquist plots exhibit a semicircle in the high-frequency range, which is related to the resistance of the surface between catalyst and electrolyte. It clearly shows that the CoP-CGP2 possesses the smallest semicircle, suggesting a faster electron transfer process on the interface of the as-prepared CoP-CGP2.

In addition, as a potential electrocatalyst with high activity for OER, the stability is also an important property to ensure that the catalyst can release a high activity for a long time (Ding et al., 2018). As shown in the inset curve of Figure 6B, chronoamperometry was conducted at an overpotential of 310 mV for CoP-CGP2. The regular peaks are the cumulated O_2_ bubble releasing. It can be observed that activity slightly dropped after 5 h. Furthermore, the stability of CoP-CGP2 was evaluated again by the CV method. As shown in Figure 6B, the LSV curves of before and after 1000 cycles present a very small change. All of these results prove that the as-prepared CoP-CGP2 shows not only high activity but also good stability. We also take our as-prepared materials into comparison with previous reported OER electrocatalysts, which is shown in Table 2. Our as-prepared materials can release a relatively high OER activity in 1 M KOH, which make them potential candidates for OER electrocatalysts. Furthermore, the HER activity was also evaluated in 1 M KOH shown in Figure S4.

**Table 2:** The OER activity comparison of recent Co-based catalysts.

**Sample**	**Electrolyte**	**Overpotential (mV)**	**References**
Co-CGP1	1 M KOH	390	This work
Co-CGP2	1 M KOH	310	This work
Co-CGP3	1 M KOH	400	This work
RuO_2_	1 M KOH	330	This work
CoP/CoP_2_@NPCNT	1 M KOH	330	Li et al., 2018a
Ni/Co-P	1 M KOH	360	Zheng et al., 2019
CoP@NG	1 M KOH	354	Lu et al., 2019
CoP_2_@3D-NPC	1 M KOH	350	Yang et al., 2019b

## Conclusion

In summary, we have successfully synthesized a novel CoP-based OER electrocatalyst by means of a facile one-step calcination strategy, where the CoP nanoparticles are encapsulated by 2D N, P-codoped carbon nanosheets. The added phosphoric acid during the synthesis process not only plays the role of phosphor source in the formation of CoP and pore-forming agent but also is doped into the N-doped carbon matrix. Benefiting from the ideal structure, the biomolecule-based 2D N, P-doped carbon nanosheets can supply well-developed porosity to promote the mass transfer and meanwhile preserve the covering CoP nanoparticles during a long-time electrocatalytic performance. As expected, the as-prepared electrocatalyst shows a superior OER activity with good stability in alkaline condition. Considering the simple synthesis strategy and high OER electrocatalytic performance, we believe that this work can not only supply a potentially competitive electrocatalyst but also be extended to design and synthesize other kinds of catalysts.

## Data Availability Statement

All datasets generated for this study are included in the article/Supplementary Material.

## Author Contributions

YL conducted the experiments and wrote the manuscript. XG and BH helped with operating the experiments and data analysis. QW and ZX supervised the research. BH and QW analyzed the electrochemical test and discussion. All authors approved the submission of the final manuscript.

### Conflict of Interest

The authors declare that the research was conducted in the absence of any commercial or financial relationships that could be construed as a potential conflict of interest.
