# Supplementary material for: One-Step Synthesis of N, P-Codoped Carbon Nanosheets Encapsulated CoP Particles for Highly Efficient Oxygen Evolution Reaction
Source: Front Chem. 2020 Jan 9;7:805. doi: 10.3389/fchem.2019.00805 (PMC6962193; doi:10.3389/fchem.2019.00805)
Supplement: Supplementary file 1 [file Data_Sheet_1.pdf]

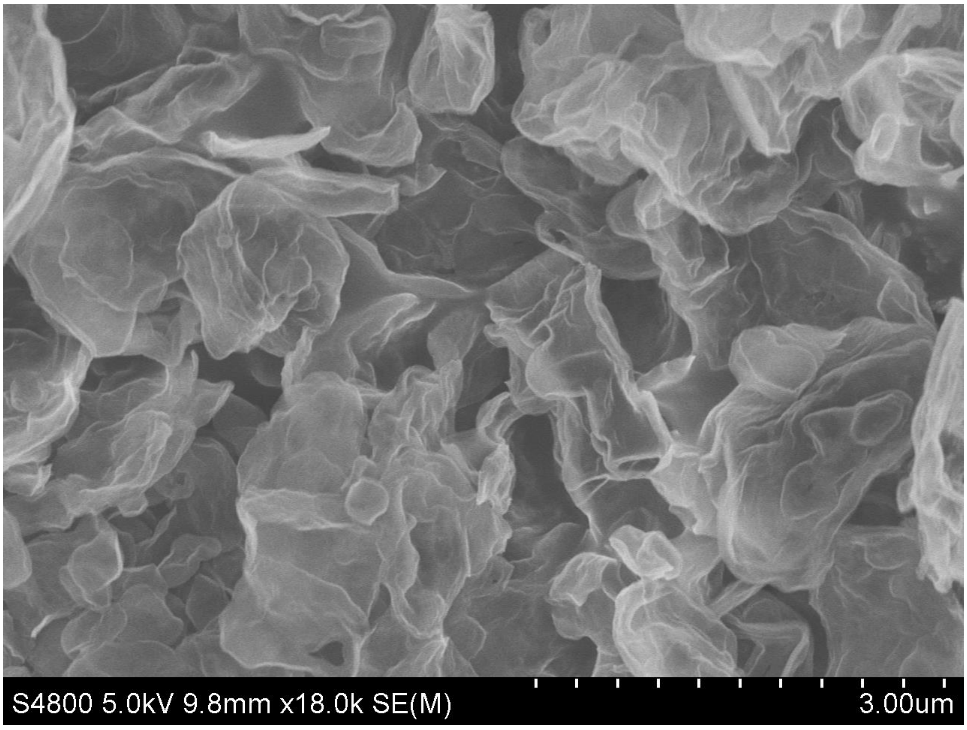

Figure S1. SEM image of CG

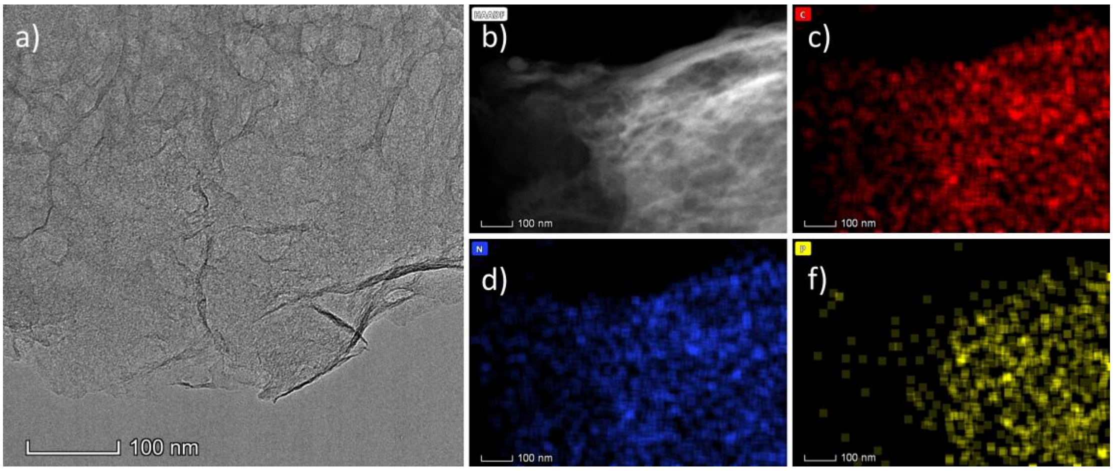

Figure S2. a) TEM of CGP; b) HAADF-STEM image; and EDS elemental mapping: c) C; d) N; f) P.

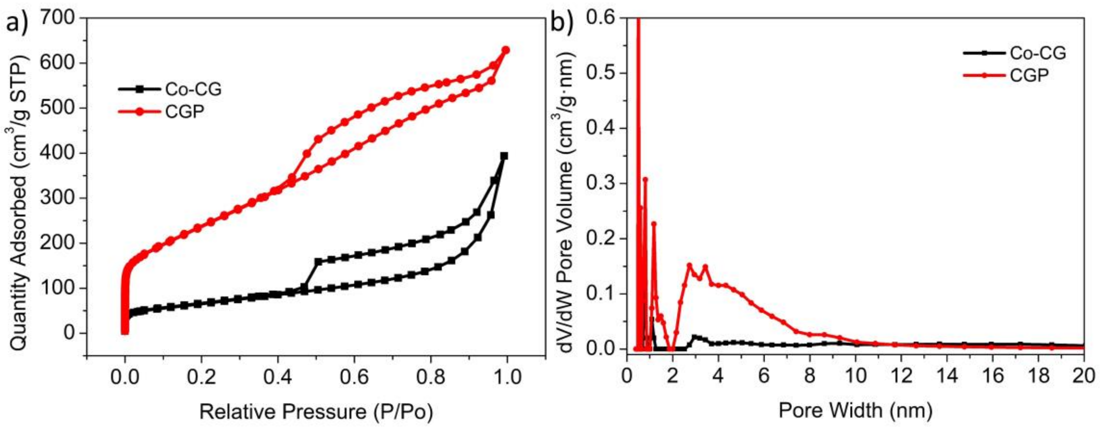

Figure S3. a) N_2 adsorption/desorption isotherms and b) pore size dispersion plots.

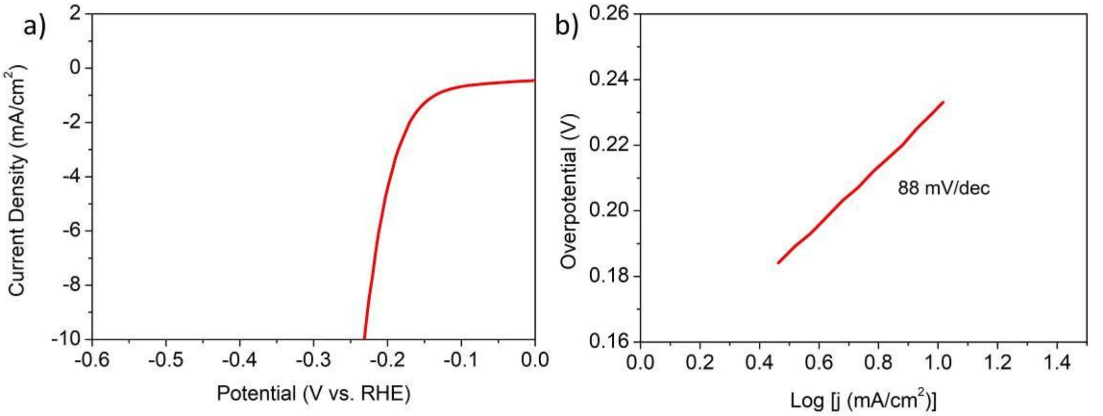

Figure S4. a) LSV curve of Co-CGP2 in 1M KOH and corresponding b) Tafel slope.
